# Synthesis and Structure Elucidation of Novel Spirooxindole Linked to Ferrocene and Triazole Systems *via* [3 + 2] Cycloaddition Reaction

**DOI:** 10.3390/molecules27134095

**Published:** 2022-06-25

**Authors:** Mezna Saleh Altowyan, Saied M. Soliman, Matti Haukka, Nora Hamad Al-Shaalan, Aminah A. Alkharboush, Assem Barakat

**Affiliations:** 1 Department of Chemistry, College of Science, Princess Nourah bint Abdulrahman University, P.O. Box 84428, Riyadh 11671, Saudi Arabia; nhalshaalan@pnu.edu.sa (N.H.A.-S.); amina84w@gmail.com (A.A.A.); 2 Department of Chemistry, Faculty of Science, Alexandria University, P.O. Box 426, Ibrahimia, Alexandria 21321, Egypt; saied1soliman@yahoo.com or; 3 Department of Chemistry, University of Jyväskylä, P.O. Box 35, FI-40014 Jyväskylä, Finland; matti.o.haukka@jyu.fi; 4 Department of Chemistry, College of Science, King Saud University, P.O. Box 2455, Riyadh 11451, Saudi Arabia

**Keywords:** spirooxindole, ferrocene, triazole, azomethine ylide, [3 + 2] cycloaddition (32CA) reaction

## Abstract

In the present work, a novel heterocyclic hybrid of a spirooxindole system was synthesized *via* the attachment of ferrocene and triazole motifs into an azomethine ylide by [3 + 2] cycloaddition reaction protocol. The X-ray structure of the heterocyclic hybrid (1″*R*,2″*S*,3*R*)-2″-(1-(3-chloro-4-fluorophenyl)-5-methyl-1*H*-1,2,3-triazole-4-carbonyl)-5-methyl-1″-(ferrocin-2-yl)-1″,2″,5″,6″,7″,7a″-hexahydrospiro[indoline-3,3″-pyrrolizin]-2-one revealed very well the expected structure, by using different analytical tools (FTIR and NMR spectroscopy). It crystallized in the triclinic-crystal system and the *P*-*1*-space group. The unit cell parameters are *a* = 9.1442(2) Å, *b* = 12.0872(3) Å, *c* = 14.1223(4) Å, *α* = 102.1700(10)°, *β* = 97.4190(10)°, *γ* = 99.1600(10)°, and V = 1484.81(7) Å^3^. There are two molecules per unit cell and one formula unit per asymmetric unit. Hirshfeld analysis was used to study the molecular packing of the heterocyclic hybrid. H···H (50.8%), H···C (14.2%), Cl···H (8.9%), O···H (7.3%), and N···H (5.1%) are the most dominant intermolecular contacts in the crystal structure. O···H, N···H, H···C, F···H, F···C, and O···O are the only contacts that have the characteristic features of short and significant interactions. AIM study indicated predominant covalent characters for the Fe–C interactions. Also, the electron density (ρ(r)) at the bond critical point correlated inversely with the Fe–C distances.

## 1. Introduction

Metal-based-drug development has been seen a tremendous interest recently in related drug-discovery programs [1,2]. In particular, sandwich metallocenes derived from transition-metal ions (such as ferrocene, osmocene, ruthenocene, titanocene, and others) have been proven to be promising lead compounds for drug discovery and development [3,4]. Among them, ferrocene is the most common and is utilized as a synthon to prepare a library of molecules with divergent functionalization in the structural framework [5,6,7]. For clinical uses, there are many representative examples of engrafted ferrocene therapeutic agents, such as ferroquine as an anti-malarial agent and hydroxyferrocifen for breast-cancer treatment [8,9]. Several studies have received a lot of attention, for constructing ferrocene grafting in an organic-compounds-molecular framework, due to the unique reactivity and diversity of biological activity [10,11,12].

As for the sequences, a technique that has a single molecular framework with different biologically active scaffolds may add adverse health issues, so this co-administration will probably enhance the pharmaceutical potential of the new hybrid skeleton. Another exciting and interesting biological activity, a pharmacophore, is the 1,2,3-triazole scaffold exhibited by many pharmaceutical targets [13]. In this regard, Supuran and co-workers designed and synthesized new ferrocene–triazole hybrids linked to benzenesulfonamides derivatives, which were subsequently evaluated as carbonic anhydrase inhibitors that showed inhibitory activity with Ki in a nanomolar scale [14]. Another representative example reported by Kumar et al., which succeeded in linking the ferrocene–triazole scaffold into the isatin motif as a lead compound for tuberculosis treatment [15,16]. Another generation has been reported by the same group, which functionalized an isatin- and ferrocene-based triazole that exhibited biological activity as an anti-malarial agent [17]. The system of congugated ferrocene–triazole has many potential applications in host–guest chemistry, conducting polymers, nanoscience, sensing, and electrochemical detection as well as for biosensing probes in medicinal chemistry [18]. Recently, Zhao and co-workers have reported a series of ferrocene–triazole receptors tethered to quinoline or naphthalene rings, and these molecular receptors have proven to be naked-eye chemosensors and fluorescent probs for Cu^+2^ [19]. In another example, based on multisignaling sensors for Hg^+2^ towards live-cell imaging, Sundargopal Ghosh and co-workers reported the synthesis of ferrocene–triazole linked to a rhodamine system, which proved to be highly sensitive, and selective fluorescent sensors for the detection of Hg^+2^ [20].

**Figure 1:**
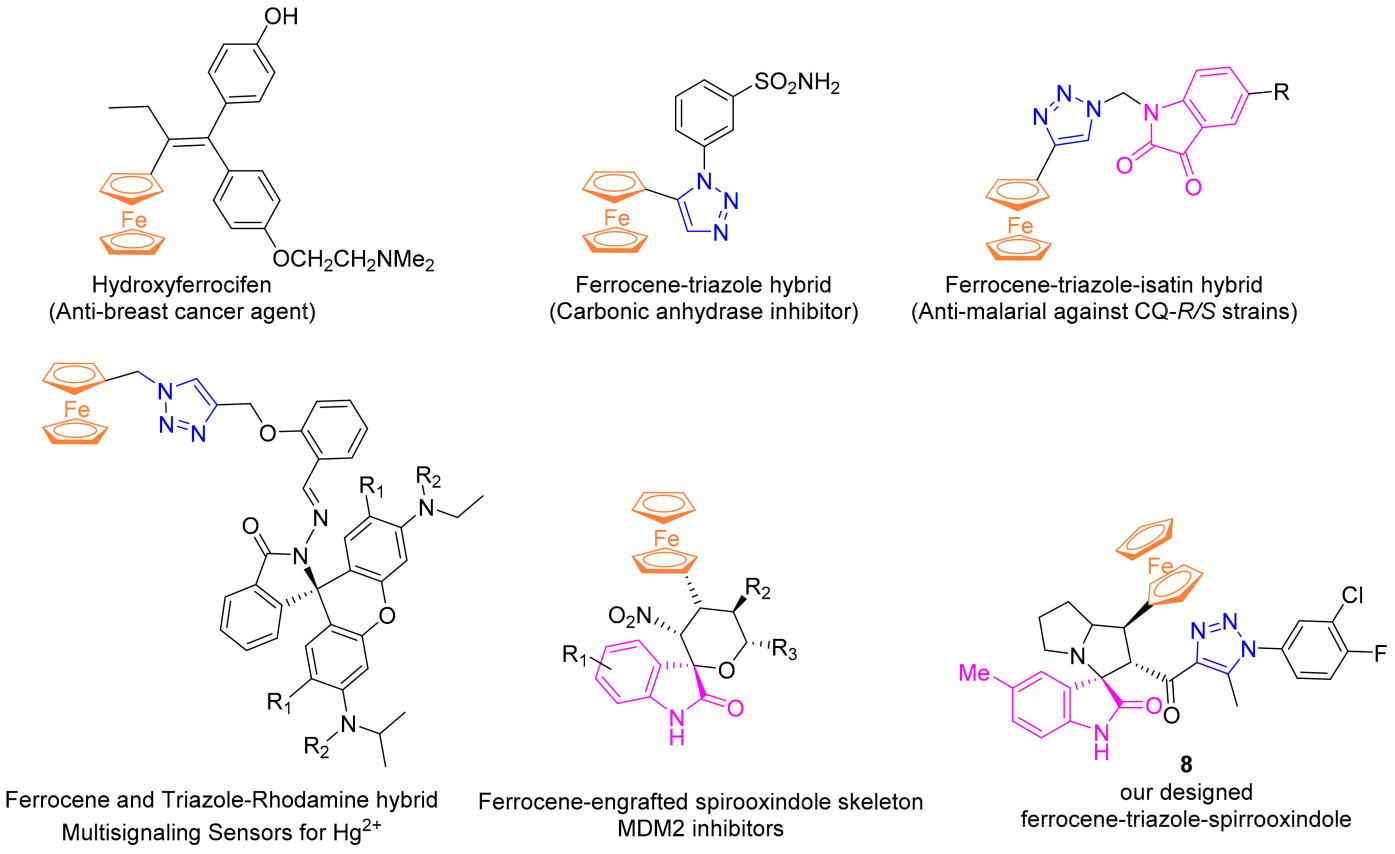
Some ferrocene/ferrocene–triazole/ferrocene–spirooxindole-based pharmacophores.

Many approaches have been reported for co-administrated ferrocene-appended biologically active hybrids, which act as anti-microbial, anti-plasmodial, anti-oxidant, anti-proliferative, anti-inflammatory, and anti-tubercular, with many other applications [21,22]. However, ferrocene-insertion strategies rarely focus on the drug skeleton for the design of new derivatives of spirooxindole, a classical anticancer drug [23,24,25,26]. Wei Huang and the Gu He research group have reported an impressive hybrid containing ferrocene grafted with the spirooxindole skeleton, which has proven to be a novel MDM2 inhibitor (Figure 1) [27].

Based on this finding, and in continuation of our research program towards the synthesis of a multi-functionalized spirooxindole drug skeleton for drug-research development [28,29,30,31,32], we reported here the novel spirooxindole system appending the ferrocene–triazole–oxindole systems. The molecular and supramolecular features of this novel compound were elucidated, based on the X-ray diffraction of a single crystal, Hirshfeld analysis, and atoms-in-molecules (AIM) calculations.

## 2. Results and Discussion

### 2.1. Chemistry

**Scheme 1:**
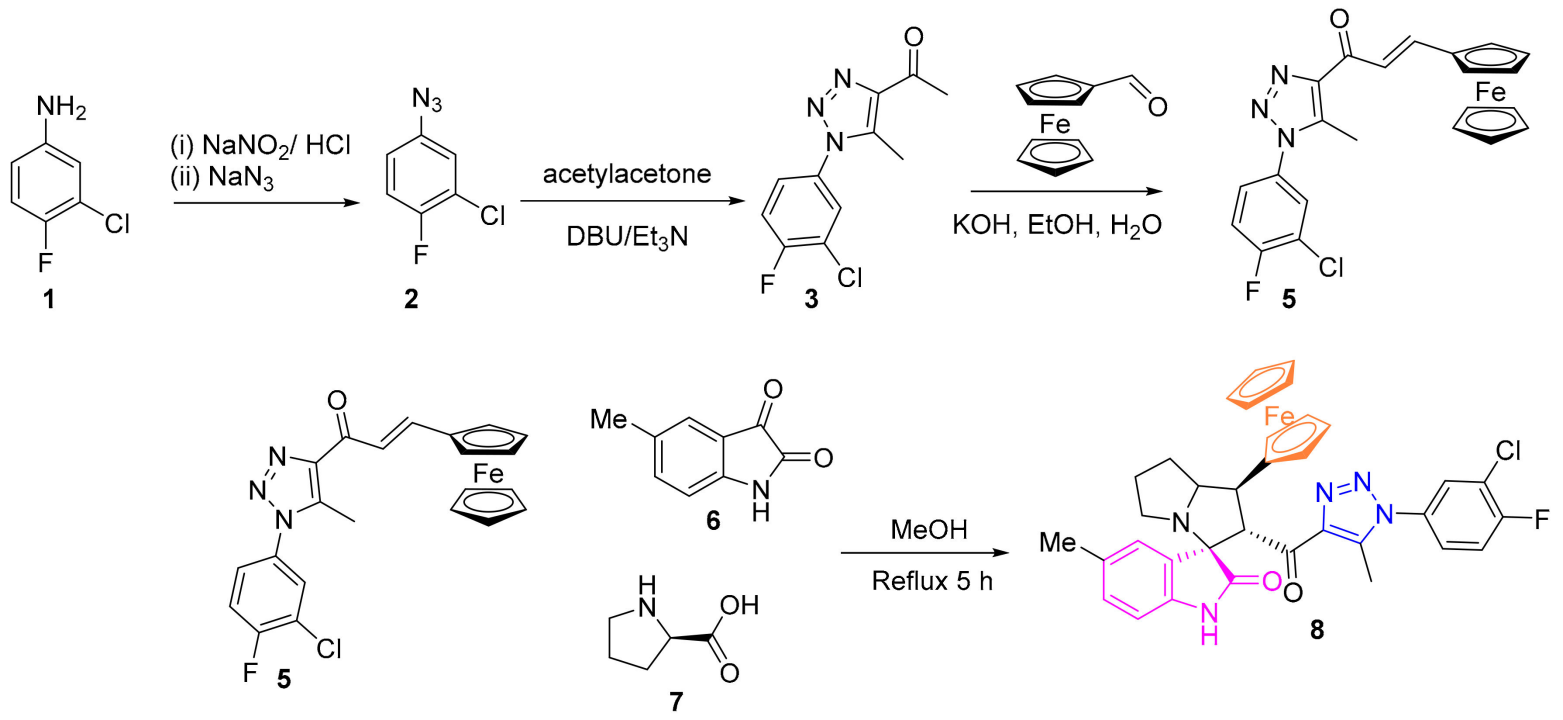
Synthetic route for the novel spirooxindole engrafted with ferrocene and triazole nucleus **8**.

**Scheme 2:**
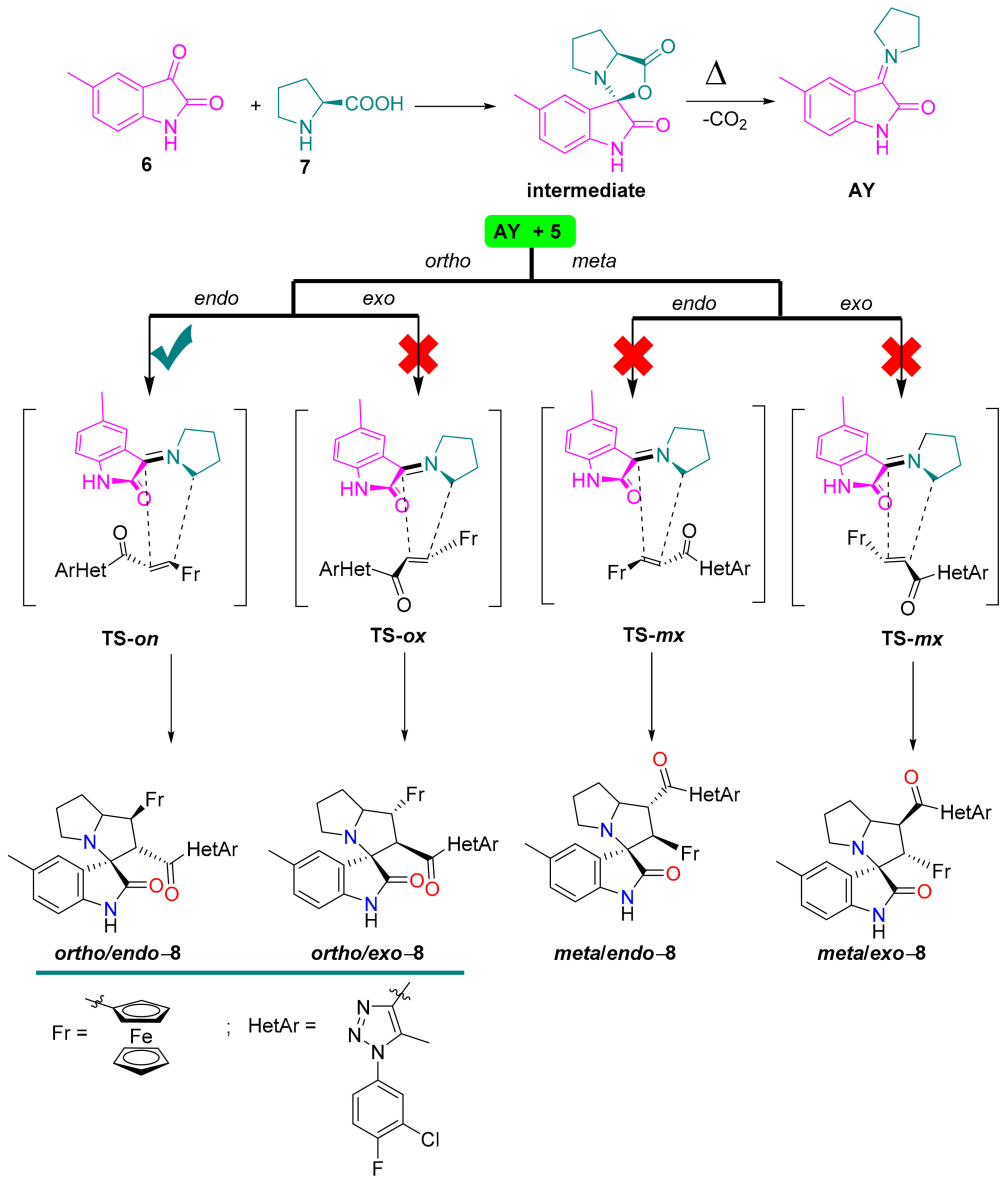
Proposed approach of AY to ethylene derivative **5**, explaining the regio- and stereoselective synthesis of **8**.

A novel, functionalized spirooxindole derivative linked to the ferrocene and triazole systems was synthesized, as shown in Scheme 1. The ethylene derivative **5** required for the [3 + 2] cycloaddition reaction was synthesized in consequential steps from 3-chloro-4-fluoroaniline, which is commercially available. The first step is to form the azide derivative, followed by a cycloaddition reaction to afford the acetyl-triazole derivative, and, finally, aldol condensation is employed to obtain the ethylene derivative having the new pharmacologically interesting hybrids. Ethylene derivative **5** was subjected into the [3 + 2] cycloaddition reaction, with the generated azomethine ylide (AY) from the 5-methyl isatin and L-proline, in refluxing methanol for 5 h. The final compound is obtained in a high-chemical yield and stereoselective fashion. The chemical architecture was assigned, based on single-crystal X-ray-diffraction analysis and a set of spectroscopic tools, including NMR and IR spectra. The plausible mechanism is depicted in Scheme 2, based on the previously reported literatures [33,34,35,36,37,38,39].

### 2.2. Crystal-Structure Description of ***8***

**Figure 2:**
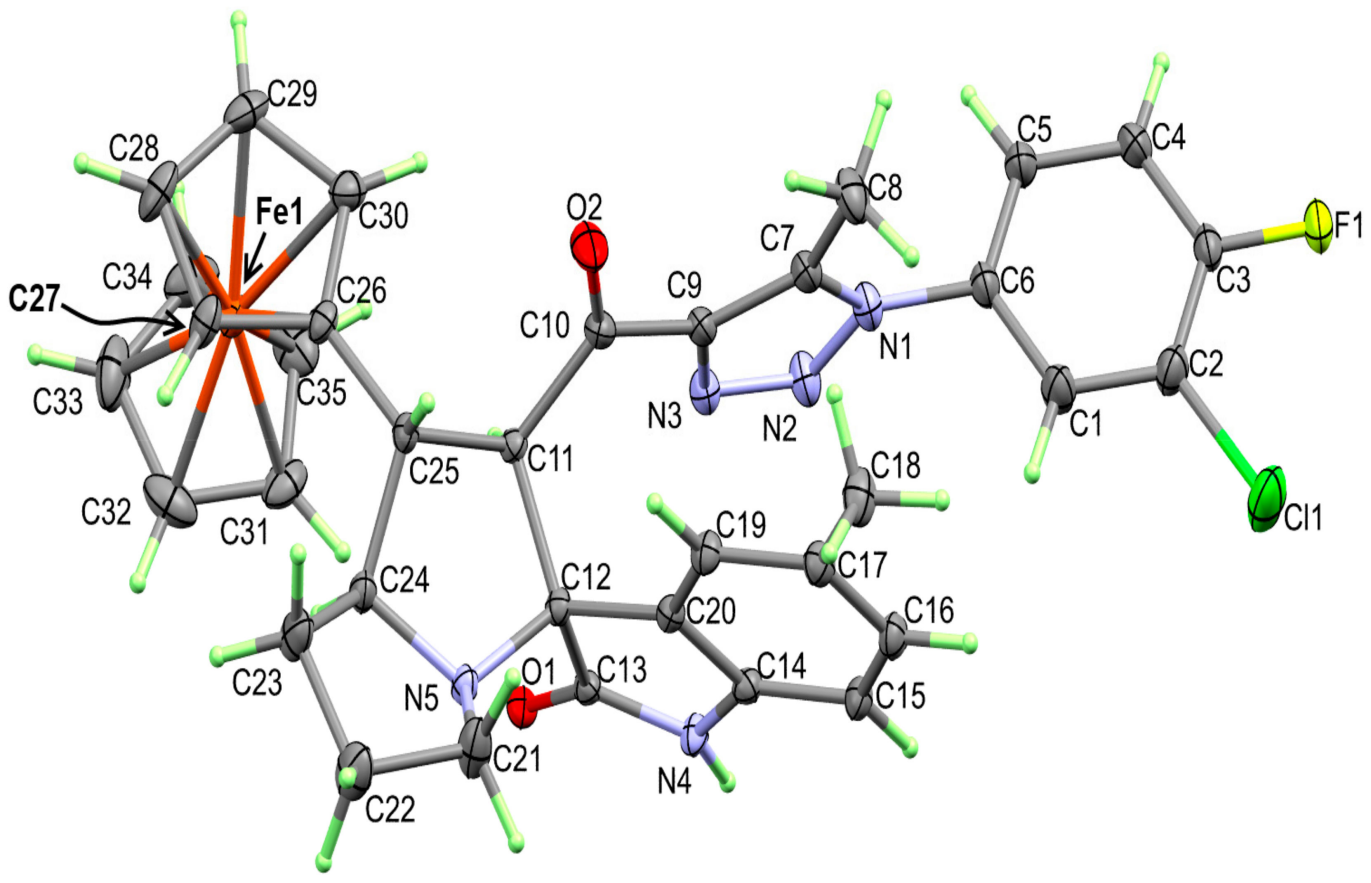
Thermal ellipsoids at 30% probability level, showing atom numbering of **8**.

**Figure 3:**
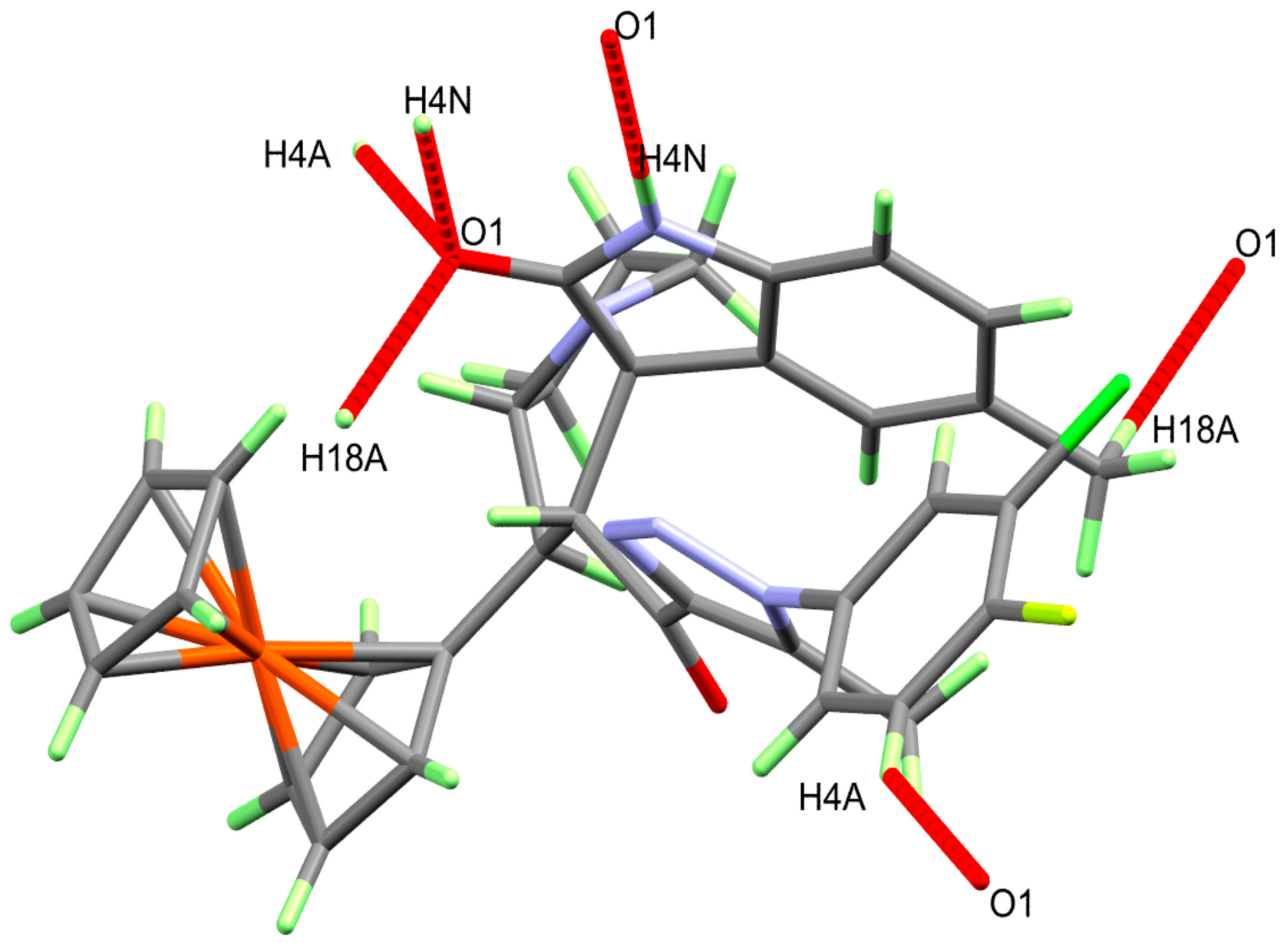
The most important intermolecular contacts in the crystal structure of **8**.

The X-ray structure of **8** confirmed very well the structure of the target product (Figure 2), and the crystallographic details are summarized in Table 1. It crystallized in the triclinic-crystal system and the *P*-*1*-space group, with z = 2 and one molecular unit as the asymmetric formula. The unit cell parameters are *a* = 9.1442(2) Å, *b* = 12.0872(3) Å, *c* = 14.1223(4) Å, *α* = 102.1700(10)°, *β* = 97.4190(10)°, *γ* = 99.1600(10)°, and V = 1484.81(7) Å^3^. The list of bond distances and angles is given in Table 2. The structure comprised a ferrocene moiety connected with a pyrrolizine fragment, which is considered as a part of the spiro structure with the indolone moiety and is also connected with the triazole moiety via the carbonyl C10 = O2 group (Figure 3).

**Figure 4:**
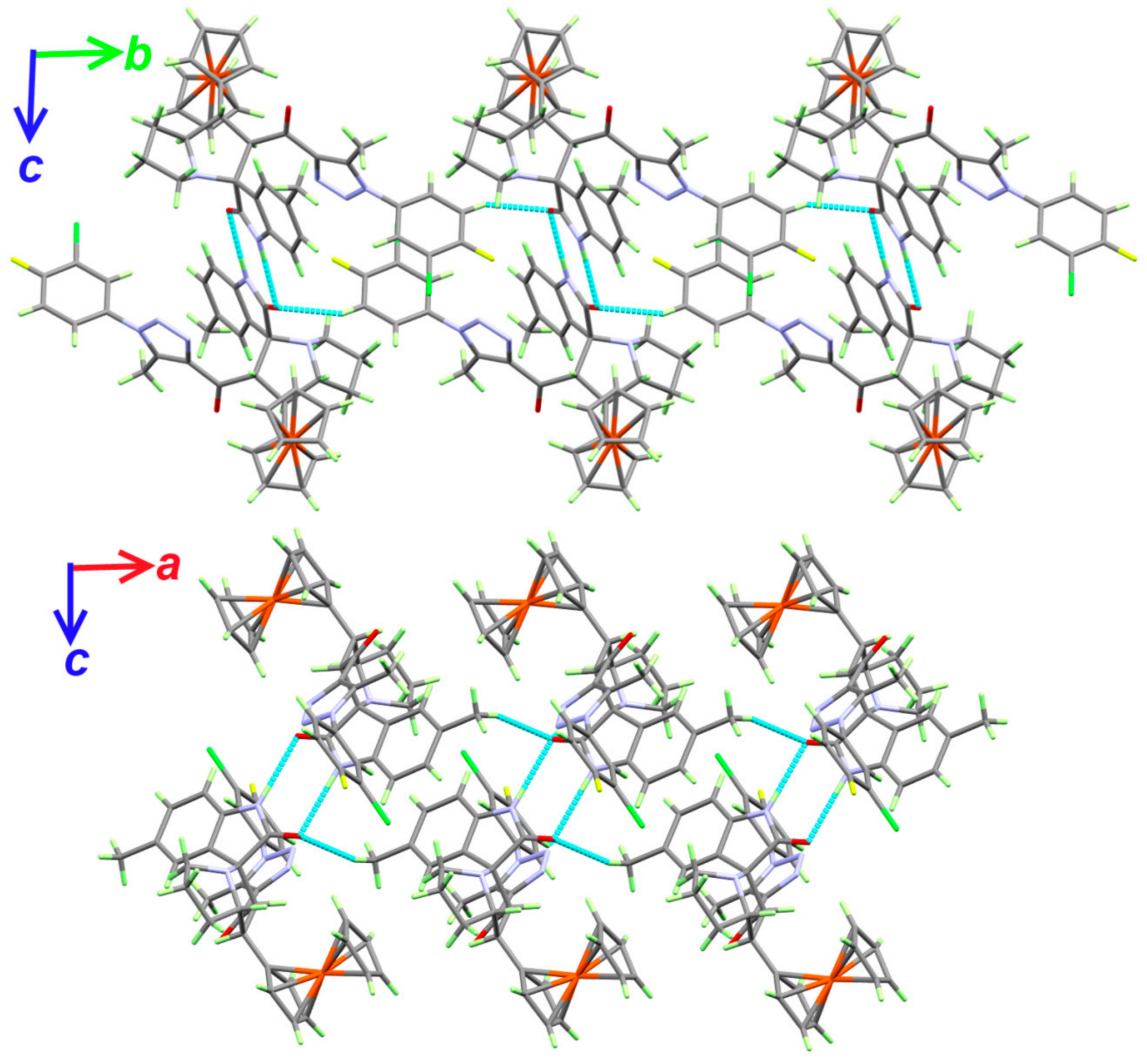
Packing of the molecular units along *ac* and *bc* planes in **8**.

The supramolecular structure of **8** is controlled by the two intermolecular interactions depicted in Table 3 and is shown as a red dotted line in Figure 2. The H···O interaction distances are 2.05(2) and 2.22 Å for N4-H4N···O1 and C4-H4A···O1, respectively. Views along the *ac* and *bc* planes for the packing structures are shown in Figure 4.

### 2.3. Hirshfeld Surface Analysis

**Figure 5:**
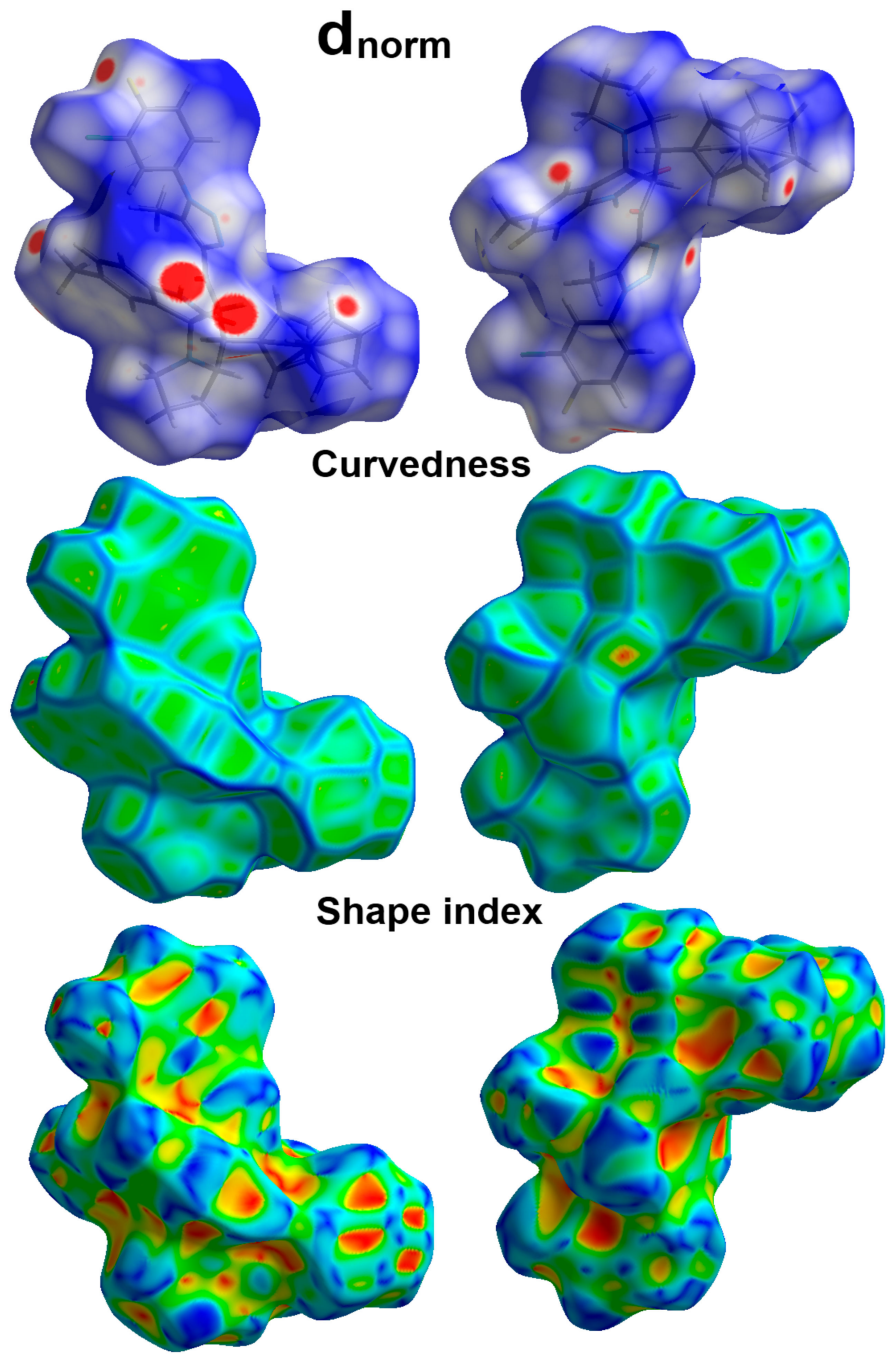
Hirshfeld surfaces of **8**.

Crystal structure is built from the aggregation of molecular fragments via a definite set of intermolecular contacts, which hold these fragments in a certain organization in the crystal. Hirshfeld analysis is an important tool to detect all the intermolecular contacts, both at the qualitative and quantitative levels. There are three Hirshfeld surfaces, which are d_norm_, shape index, and curvedness maps (Figure 5). The d_norm_ map sheds light on all intermolecular contacts in the form of red, white, and blue regions, which indicate shorter, equal, and longer interaction distances, respectively, than the vdWs radii sum of the interacting atoms. Shape index and curvedness maps with blue/red triangles and a green, flat area, respectively, are indicators of π–π stacking interactions.

**Figure 6:**
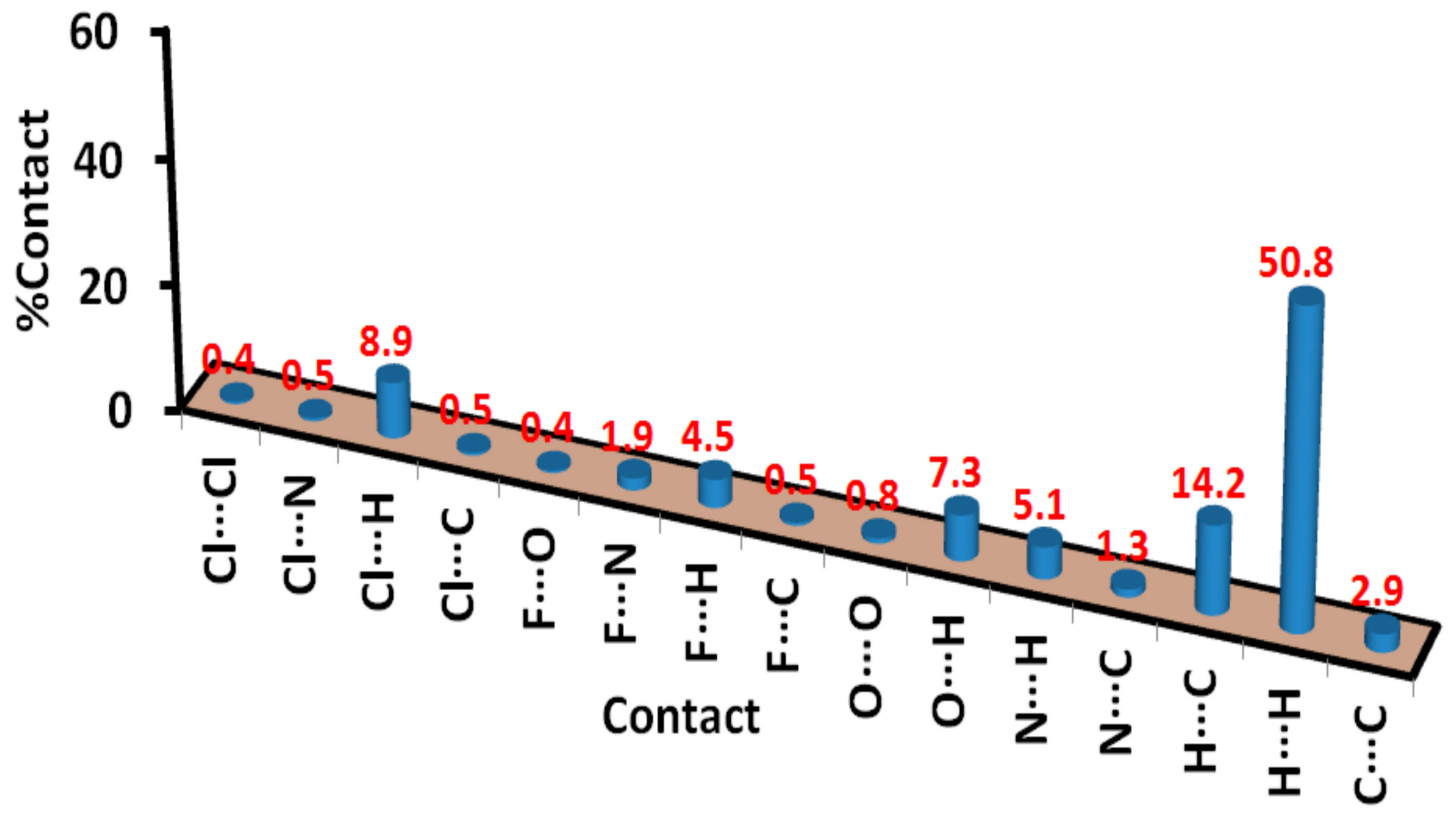
Intermolecular contacts and their percentages in **8**.

**Figure 7:**
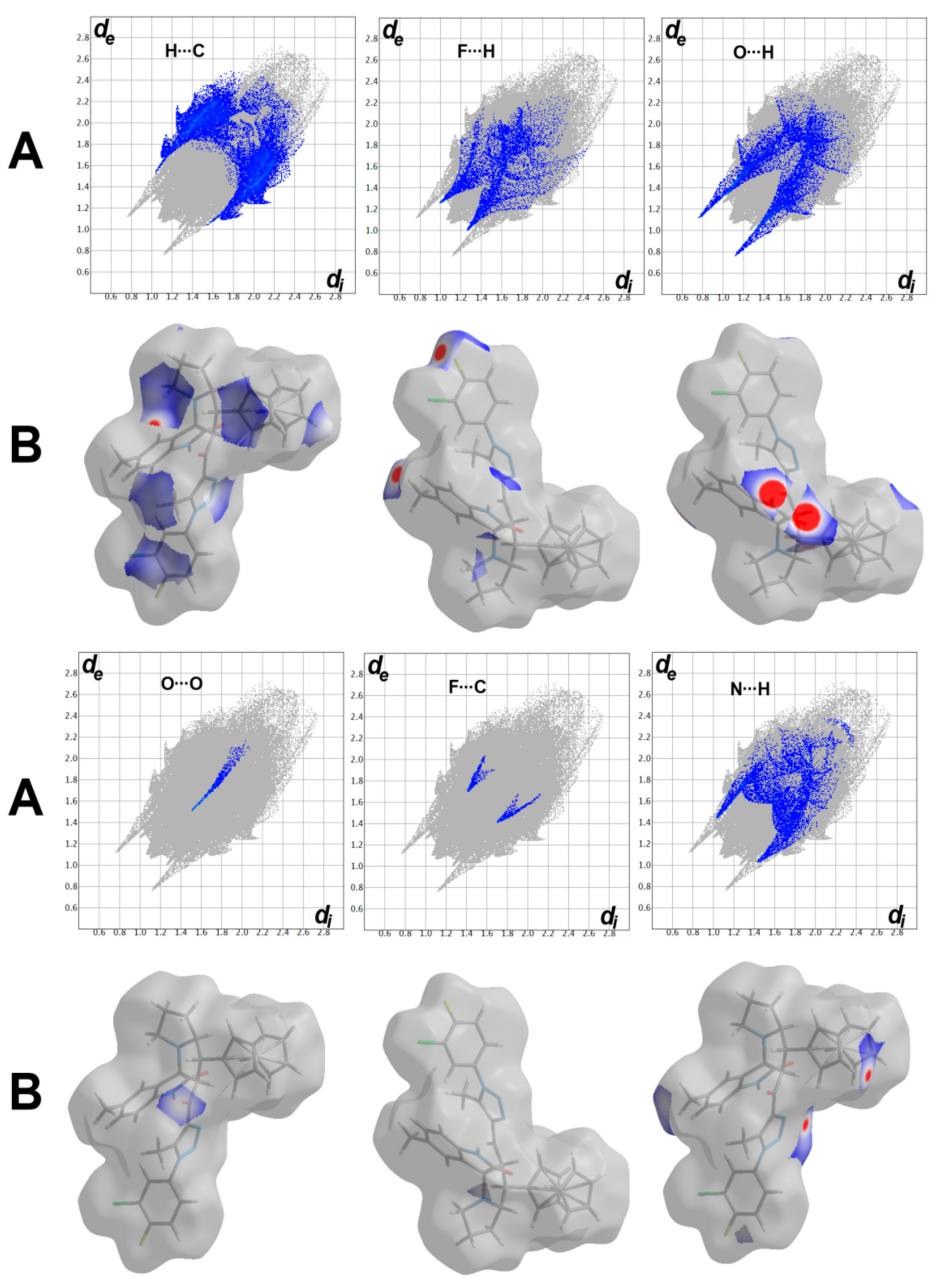
Fingerprint plots (**A**) and decomposed d_norm_ maps (**B**) of short contacts in **8**.

Many short contacts were detected from the analysis of the d_norm_ map. With the aid of a fingerprint, the different intermolecular contacts were predicted. It is found that the H···H (50.8%), H···C (14.2%), Cl···H (8.9%), O···H (7.3%), and N···H (5.1%) contacts are the most dominant. The percentages of all possible contacts in the crystal structure of 8 are shown in Figure 6. Careful inspection of the d_norm_ map revealed the appearance of the O···H, N···H, H···C, F···H, F···C, and O···O contacts as red spots, as shown in Figure 7. In the same figure, the decomposed fingerprint plots clearly show the characteristics of the sharp spikes for the short-distance interactions. All these short contacts, along with their corresponding interaction distances, are listed in Table 4.

It is worth noting that there is one short H···H interaction that appeared in the d_norm_ as a white region. The corresponding H16···H21B contact distance is 2.168 Å, while twice the vdWs radii of hydrogen is 2.18 Å. Hence, H16···H21B is slightly shorter than this value. Analysis of shape index and curvedness maps revealed the absence of the main features of the π–π stacking interactions in this system. Other interactions are considered weak interactions and have less contributions in the molecular packing of **8**.

### 2.4. AIM Analysis

**Figure 8:**
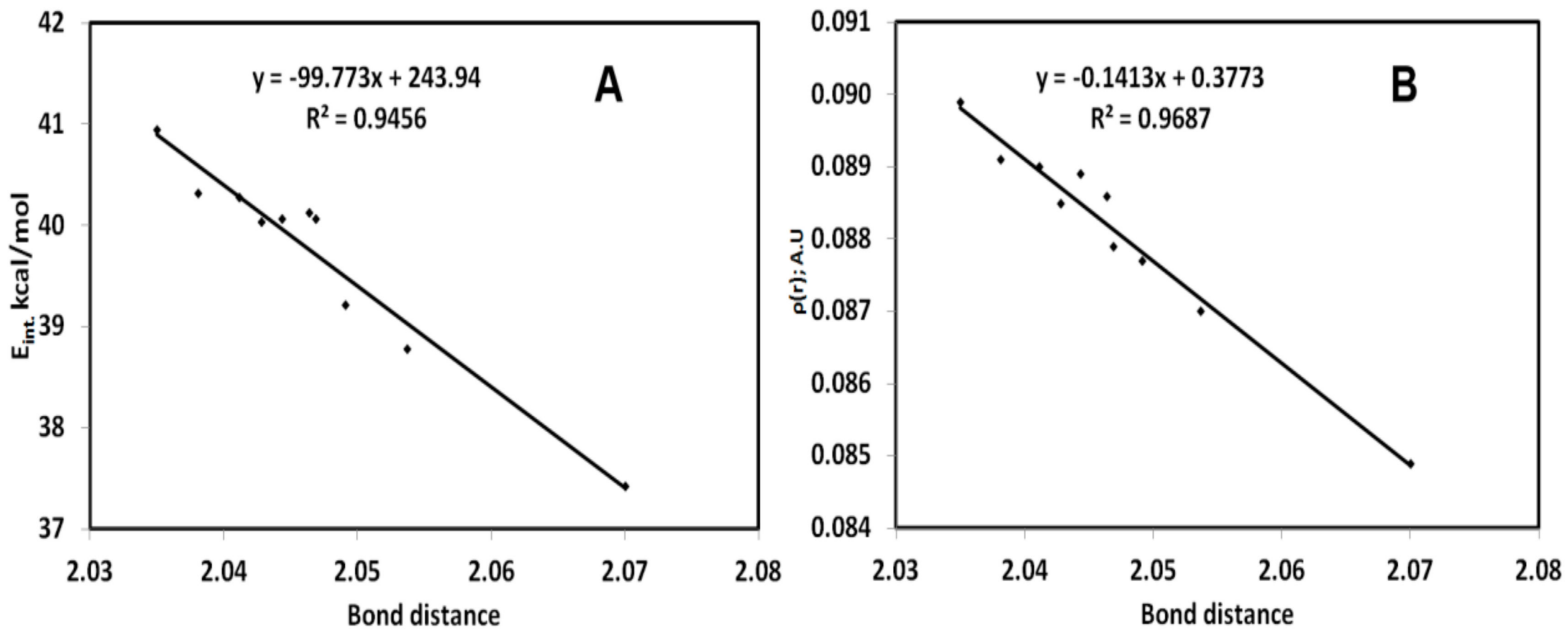
Correlations between bond distances, and ρ(r) (**A**) and E_int._ (**B**).

The atoms-in-molecules (AIM)-topology parameters have interesting applications in deciding the nature and strength of atom–atom interactions [40,41,42,43,44,45,46,47,48]. In this regard, nature and strength of the Fe–C interactions in the ferrocenyl moiety were elucidated using the AIM parameters listed in Table 5. The electron density (ρ(r)) values are close to 0.1 a.u. and were found to be inversely correlated to the Fe–C distances (Figure 8A). As a result, short-distance interactions are characterized by high electron density at the bond critical point, where stronger bonds have greater ρ(r) values than weaker ones. In agreement with this conclusion, the interaction energies (E_int_) of the Fe–C bonds are also found in good correlation with the Fe–C distances (Figure 8B). The correlation coefficients (R^2^) for ρ(r) and E_int_ are 0.9456 and 0.9687, respectively. On the other hand, the total energy density (H(r)) and ratio of the potential to kinetic energy density (V(r)/G(r)) were employed to predict the nature of the Fe–C interactions. The H(r) with a negative value and V(r)/G(r) > 1 are indicators of the predominant covalent characters for these bonds.

## 3. Materials and Methods

All details regarding instruments and chemicals used in this study are provided in the Supplementary Materials. The synthesis of 1-(1-(3-chloro-4-fluorophenyl)-5-methyl-1*H*-1,2,3-triazol-4-yl)ethan-1-one **3** followed the reported procedure [49].

### 3.1. Synthesis of (E)-1-(1-(3-Chloro-4-fluorophenyl)-5-methyl-1H-1,2,3-triazol-4-yl)-3-(ferrocin -2-yl)-prop-2-en-1-one ***5***

A mixture of ferrocene carboxyladehyde (2.0 mmol, 428 mg) and 1-(1-(3-chloro-4-fluorophenyl)-5-methyl-1*H*-1,2,3-triazol-4-yl)ethan-1-one 3 (0.506 g, 2.0 mmol) dissolved in ethanol (20 mL) was added slowly to an aqueous solution of potassium hydroxide (2.0 mmol, 112 mg) in water (10 mL). The mixture was stirred in crushed-ice bath for 2 h then stirred at 20~25 °C for 4 h. The mixture was filtrated and the residue was washed with cold water and cold alcohol then dried to give the titled compound without further purification.

^1^ H NMR (400 MHz, CDCl_3_) δ 7.87 (d, *J* = 15.4 Hz, 1H), 7.62 (d, *J* = 20.6 Hz, 1H), 7.37 (s, 1H), 4.68 (s, 1H), 4.51 (s, 1H), 4.18 (s, 3H), 2.65 (s, 3H). ^13^ C NMR (101 MHz, CDCl_3_) δ 183.64, 160.19, 157.67, 144.31, 138.41, 132.04, 127.91, 125.41, 122.83, 122.64, 117.82, 79.24, 70.14. Chemical Formula: C_22_H_17_ClFFeN_3_O.

### 3.2. Synthesis of (1′R,2′S,3R)-2′-(1-(3-Chloro-4-fluorophenyl)-5-methyl-1H-1,2,3-triazole-4- carbonyl)-5-methyl-1′-(ferrocin-2-yl)-1′,2′,5′,6′,7′,7a′-hexahydrospiro[indoline-3,3′-pyrrolizin]-2-one ***8***

A mixture of **5** (157.15 mg, 0.35 mmol), 5-Me-isatin (56.36 mg, 0.35 mmol), and L-proline (40.25 mg, 0.35 mmol) in methanol (10 mL) was refluxed on oil bath for appropriate time 5–8 h. After completion of the reaction as evident from TLC, the reaction was kept at room temperature overnight and the solid precipitate was filtered off without further purification. Crystalline compound obtained by slow evaporation in methanol.

^1^ H NMR (400 MHz, DMSO-*d*_6_) δ 10.08 (s, 1H), 7.80 (s, 1H), 7.71 (s, 1H), 7.45 (s, 1H), 6.82 (s, 2H), 6.45 (s, 1H), 5.12 (s, 1H), 4.26 (s, 5H), 4.11 (s, 3H), 3.82 (s, 1H), 2.76 (s, 1H), 2.35 (s, 0H), 2.15 (s, 3H), 2.07 (s, 2H), 1.98 (s, 1H), 1.77 (s, 2H); ^13^ C NMR (101 MHz, DMSO-*d*_6_) δ 194.07, 180.08, 160.72, 155.74, 143.49, 140.68, 138.45, 132.23, 130.42, 129.81, 129.05, 126.97, 126.09, 119.55, 118.07, 108.68, 108.20, 90.04, 72.13, 69.82, 68.08, 67.28, 66.74, 65.95, 43.53, 9.30.; IR IR (KBr, cm^−1^): 3250, 3090, 2825, 1730, 1667, 1620, 1595, 1535, 1502, 1469, 1394, 1328, 1287, 1220; Chemical Formula: C_35_H_31_ClFFeN_5_O_2_.

### 3.3. X-ray Structure Determinations

The crystal of **8** was immersed in cryo-oil, mounted in a loop, and measured at a temperature of 170 K. The X-ray-diffraction data were collected on a Bruker Kappa Apex II diffractometer using Mo Kα radiation. The *Denzo-Scalepack* [50] software package was used for cell refinement and data reduction. A multi-scan absorption correction based on equivalent reflections (*SADABS* [51]) was applied to the intensities before structure solution. The structure was solved by intrinsic phasing method using *SHELXT* [52] software. Structural refinement was carried out using *SHELXL* [53] software with *SHELXLE* [54] graphical user interface. The NH hydrogen atom was located from the difference Fourier map and refined isotropically. Other hydrogen atoms were positioned geometrically and constrained to ride on their parent atoms, with C-H = 0.95–1.00 Å and U_iso_ = 1.2–1.5 U_eq_ (parent atom).

### 3.4. Hirshfeld Surface Analysis

The topology analyses were performed using Crystal Explorer 17.5 program [55].

### 3.5. DFT Calculations

The MPW1PW91/TZVP method [56] with the aid of Gaussian 09 software [57] was used to perform single-point calculations to compute the atoms-in-molecules (AIM) parameters with the aid of Multiwfn program [58].

## 4. Conclusions

Novel spirooxindole-incroporated ferrocene–triazole scaffolds was synthesized by 32CA *via ortho*/*endo* pathway. The structure of **8** is further confirmed using a single-crystal X-ray structure, and the results were used to analyze its supramolecular structure with the aid of Hirshfeld analysis. The results revealed the significance of the O···H, N···H, H···C, F···H, F···C, and O···O contacts in the molecular packing of **8**. DFT calculations employing atoms in molecular parameters were used to investigate the nature of the Fe–C interactions in the ferrocenyl moiety. The results shed light on the covalent character of the Fe–C-coordination interactions. Further study about this novel ferrocene–triazole based spirooxindole is underway in our laboratory. 

## Figures and Tables

**Table 1:** Crystal data.

	8
CCDC	2164622
empirical formula	C_35_H_31_ClFFeN_5_O_2_
fw	663.95
temp (K)	170(2)
*λ* (Å)	0.71073
cryst syst	Triclinic
space group	P $\bar{1}$
*a* (Å)	9.1442(2)
*b* (Å)	12.0872(3)
*c* (Å)	14.1223(4)
α(deg)	102.1700(10)
β (deg)	97.4190(10)
γ(deg)	99.1600(10)
*V* (Å^3^)	1484.81(7)
Z	2
*ρ*_calc_ (Mg/m^3^)	1.485
*μ*(Mo K*α*) (mm^−1^)	0.647
No. reflns.	25,941
Unique reflns.	7033
Completeness to θ = 25.242°	98.8%
GOOF (*F*^2^)	1.069
R_int_	0.0309
*R*_1_^a^ (*I* ≥ 2σ)	0.0398
*wR*_2_^b^ (*I* ≥ 2σ)	0.0916

^a^*R*_1_ = Σ||*F*_o_| − |*F*_c_||/Σ|*F*_o_|. ^b^ *wR*_2_ = {Σ[*w*(*F*_o_^2^ − *F*_c_^2^)^2^]/Σ[*w*(*F*_o_^2^)^2^]}^1/2^.

**Table 2:** Selected bond lengths [Å] and angles [°] for **8**.

**Atoms**	**Distance**	**Atoms**	**Distance**
Fe(1)-C(35)	2.035(2)	O(2)-C(10)	1.211(2)
Fe(1)-C(29)	2.038(2)	N(1)-C(7)	1.349(2)
Fe(1)-C(33)	2.042(2)	N(1)-N(2)	1.368(2)
Fe(1)-C(28)	2.043(2)	N(1)-C(6)	1.435(2)
Fe(1)-C(32)	2.045(2)	N(2)-N(3)	1.301(2)
Fe(1)-C(31)	2.046(2)	N(3)-C(9)	1.362(2)
Fe(1)-C(34)	2.047(2)	N(4)-C(13)	1.349(2)
Fe(1)-C(27)	2.049(2)	N(4)-C(14)	1.404(2)
Fe(1)-C(30)	2.0536(19)	N(5)-C(21)	1.465(2)
Fe(1)-C(26)	2.0699(18)	N(5)-C(12)	1.471(2)
F(1)-C(3)	1.341(2)	N(5)-C(24)	1.479(2)
O(1)-C(13)	1.230(2)		
**Atoms**	**Angle**	**Atoms**	**Angle**
C(35)-Fe(1)-C(29)	118.80(10)	C(33)-Fe(1)-C(26)	157.63(11)
C(35)-Fe(1)-C(33)	67.18(11)	C(28)-Fe(1)-C(26)	68.65(8)
C(29)-Fe(1)-C(33)	124.10(10)	C(32)-Fe(1)-C(26)	122.53(9)
C(35)-Fe(1)-C(28)	153.38(10)	C(31)-Fe(1)-C(26)	109.22(8)
C(29)-Fe(1)-C(28)	40.52(10)	C(34)-Fe(1)-C(26)	161.48(10)
C(33)-Fe(1)-C(28)	107.94(10)	C(27)-Fe(1)-C(26)	40.58(7)
C(35)-Fe(1)-C(32)	67.42(10)	C(30)-Fe(1)-C(26)	40.63(8)
C(29)-Fe(1)-C(32)	162.62(11)	C(28)-Fe(1)-C(30)	68.42(9)
C(33)-Fe(1)-C(32)	40.79(11)	C(32)-Fe(1)-C(30)	156.20(10)
C(28)-Fe(1)-C(32)	127.13(11)	C(31)-Fe(1)-C(30)	120.46(9)
C(35)-Fe(1)-C(31)	40.09(10)	C(34)-Fe(1)-C(30)	123.83(10)
C(29)-Fe(1)-C(31)	154.03(10)	C(27)-Fe(1)-C(30)	68.23(9)
C(33)-Fe(1)-C(31)	67.95(10)	C(35)-Fe(1)-C(26)	125.94(9)
C(28)-Fe(1)-C(31)	164.82(11)	C(29)-Fe(1)-C(26)	68.59(8)
C(32)-Fe(1)-C(31)	40.32(10)	C(7)-N(1)-N(2)	111.80(15)
C(35)-Fe(1)-C(34)	39.90(10)	C(7)-N(1)-C(6)	128.98(16)
C(29)-Fe(1)-C(34)	105.86(9)	N(2)-N(1)-C(6)	119.11(16)
C(33)-Fe(1)-C(34)	39.91(12)	N(3)-N(2)-N(1)	106.55(15)
C(28)-Fe(1)-C(34)	119.37(9)	N(2)-N(3)-C(9)	109.21(15)
C(32)-Fe(1)-C(34)	67.73(10)	C(13)-N(4)-C(14)	111.39(14)
C(31)-Fe(1)-C(34)	67.57(9)	C(21)-N(5)-C(12)	120.81(14)
C(35)-Fe(1)-C(27)	163.87(9)	C(21)-N(5)-C(24)	108.44(14)
C(29)-Fe(1)-C(27)	68.40(9)	C(12)-N(5)-C(24)	110.05(13)
C(33)-Fe(1)-C(27)	122.39(11)	C(6)-C(1)-C(2)	118.90(19)
C(28)-Fe(1)-C(27)	40.91(8)	C(3)-C(2)-C(1)	119.29(18)
C(32)-Fe(1)-C(27)	110.32(10)	C(3)-C(2)-Cl(1)	120.33(16)
C(31)-Fe(1)-C(27)	127.83(9)	C(1)-C(2)-Cl(1)	120.38(17)
C(34)-Fe(1)-C(27)	155.58(10)	F(1)-C(3)-C(4)	119.13(18)
C(35)-Fe(1)-C(30)	107.00(9)	F(1)-C(3)-C(2)	118.96(18)
C(29)-Fe(1)-C(30)	40.80(8)	C(4)-C(3)-C(2)	121.90(18)
C(33)-Fe(1)-C(30)	160.55(11)	C(3)-C(4)-C(5)	118.99(19)

**Table 3:** Hydrogen bonds for **8** [Å and °].

D-H…A	d(D-H)	d(H…A)	d(D…A)	<(DHA)
N(4)-H(4N)…O(1)#1	0.84(2)	2.05(2)	2.8889(19)	178(2)
C(4)-H(4A)…O(1)#2	0.95	2.44	3.254(2)	144.2

Symm. codes: #1 −x + 2,−y + 1,−z + 2 #2 x,y + 1,z.

**Table 4:** Short contacts and their distances (Å).

Contact	Distance	Contact	Distance
O1···H18A	2.494	C29···H18B	2.766
O1···H4N	1.880	H16···H21B	2.168
O1···H4A	2.330	O2···O2	3.029
N3···H29	2.468	F1···H16	2.515
C35···H19	2.571	F1···H18C	2.261
C7···H34	2.779	C13···F1	3.121

**Table 5:** AIM topological parameters (a.u.) for the Fe–C interactions.

Bond	ρ(r)	G(r)	V(r)	E_int_^a^	H(r)	V(r)/G(r)
**Fe**–**CP ^a^**						
Fe1-C26	0.0849	0.0927	−0.1193	37.4309	−0.0266	1.2869
Fe1-C27	0.0877	0.0962	−0.1250	39.2193	−0.0287	1.2994
Fe1-C28	0.0885	0.0988	−0.1276	40.0351	−0.0288	1.2915
Fe1-C29	0.0891	0.0991	−0.1285	40.3175	−0.0294	1.2967
Fe1-C30	0.0870	0.0955	−0.1236	38.7801	−0.0281	1.2942
**Fe**–**CP ^b^**						
Fe1-C31	0.0886	0.0991	−0.1279	40.1292	−0.0287	1.2906
Fe1-C32	0.0889	0.0985	−0.1277	40.0665	−0.0292	1.2964
Fe1-C33	0.0890	0.0992	−0.1284	40.2861	−0.0292	1.2944
Fe1-C34	0.0879	0.0999	−0.1277	40.0665	−0.0278	1.2783
Fe1-C35	0.0899	0.1008	−0.1305	40.9450	−0.0297	1.2946

## Data Availability

The data presented in this study are not available on request from the corresponding author.

## Supplementary Materials

The following supporting information can be downloaded at: https://www.mdpi.com/article/10.3390/molecules27134095/s1. CheckCIF/PLATON report file.
