# Supplementary material for: Synthesis and Structure Elucidation of Novel Spirooxindole Linked to Ferrocene and Triazole Systems via [3 + 2] Cycloaddition Reaction
Source: Molecules. 2022 Jun 25;27(13):4095. doi: 10.3390/molecules27134095 (PMC9268063; doi:10.3390/molecules27134095)

checkCIF/PLATON report

Structure factors have been supplied for datablock(s) ab1401

THIS REPORT IS FOR GUIDANCE ONLY. IF USED AS PART OF A REVIEW PROCEDURE FOR PUBLICATION, IT SHOULD NOT REPLACE THE EXPERTISE OF AN EXPERIENCED CRYSTALLOGRAPHIC REFEREE.

No syntax errors found. CIF dictionary Interpreting this report

Datablock: ab1401

Bond precision: C-C = 0.0029 A Wavelength=0.71073

Cell:	a=9.1442(2)	b=12.0872(3)	c=14.1223(4)
	alpha=102.170(1)	beta=97.419(1)	gamma=99.160(1)
Temperature:	170 K		

	Calculated	Reported
Volume	1484.81(7)	1484.81(7)
Space group	P -1	P -1
Hall group	-P 1	-P 1
Moiety formula	C35 H31 Cl F Fe N5 O2	C35 H31 Cl F Fe N5 O2
Sum formula	C35 H31 Cl F Fe N5 O2	C35 H31 Cl F Fe N5 O2
Mr	663.95	663.95
Dx,g cm-3	1.485	1.485
Z	2	2
Mu (mm-1)	0.647	0.647
F000	688.0	688.0
F000’	689.23	
h,k,lmax	12,15,18	12,15,18
Nref	7133	7033
Tmin,Tmax	0.850,0.902	0.697,0.746
Tmin’	0.818	

Correction method= # Reported T Limits: Tmin=0.697 Tmax=0.746
AbsCorr = MULTI-SCAN

Data completeness= 0.986 Theta(max)= 27.944

R(reflections)= 0.0398(5994) wR2(reflections)= 0.0991(7033)

S = 1.069 Npar= 412

The following ALERTS were generated. Each ALERT has the format
test-name_ALERT_alert-type_alert-level.
Click on the hyperlinks for more details of the test.

Alert level C

```
PLAT094_ALERT_2_C Ratio of Maximum / Minimum Residual Density ....       2.04 Report
PLAT911_ALERT_3_C Missing FCF Refl Between Thmin & STh/L=    0.600         61 Report
PLAT913_ALERT_3_C Missing # of Very Strong Reflections in FCF ....         19 Note
```

Alert level G

```
PLAT154_ALERT_1_G The s.u.'s on the Cell Angles are Equal ..(Note)      0.001 Degree
PLAT793_ALERT_4_G Model has Chirality at C11         (Centro SPGR)          R Verify
PLAT793_ALERT_4_G Model has Chirality at C12         (Centro SPGR)          S Verify
PLAT793_ALERT_4_G Model has Chirality at C24         (Centro SPGR)          S Verify
PLAT793_ALERT_4_G Model has Chirality at C25         (Centro SPGR)          R Verify
PLAT794_ALERT_5_G Tentative Bond Valency for Fe1       (II)      .       2.10 Info
PLAT883_ALERT_1_G No Info/Value for _atom_sites_solution_primary .     Please Do !
PLAT910_ALERT_3_G Missing # of FCF Reflection(s) Below Theta(Min).          2 Note
PLAT912_ALERT_4_G Missing # of FCF Reflections Above STh/L=  0.600         38 Note
PLAT941_ALERT_3_G Average HKL Measurement Multiplicity ..........         3.7 Low
PLAT978_ALERT_2_G Number C-C Bonds with Positive Residual Density.         17 Info
```

0 **ALERT level A** = Most likely a serious problem - resolve or explain
0 **ALERT level B** = A potentially serious problem, consider carefully
3 **ALERT level C** = Check. Ensure it is not caused by an omission or oversight
11 **ALERT level G** = General information/check it is not something unexpected

2 ALERT type 1 CIF construction/syntax error, inconsistent or missing data
2 ALERT type 2 Indicator that the structure model may be wrong or deficient
4 ALERT type 3 Indicator that the structure quality may be low
5 ALERT type 4 Improvement, methodology, query or suggestion
1 ALERT type 5 Informative message, check

It is advisable to attempt to resolve as many as possible of the alerts in all categories. Often the minor alerts point to easily fixed oversights, errors and omissions in your CIF or refinement strategy, so attention to these fine details can be worthwhile. In order to resolve some of the more serious problems it may be necessary to carry out additional measurements or structure refinements. However, the purpose of your study may justify the reported deviations and the more serious of these should normally be commented upon in the discussion or experimental section of a paper or in the "special_details" fields of the CIF. checkCIF was carefully designed to identify outliers and unusual parameters, but every test has its limitations and alerts that are not important in a particular case may appear. Conversely, the absence of alerts does not guarantee there are no aspects of the results needing attention. It is up to the individual to critically assess their own results and, if necessary, seek expert advice.

Publication of your CIF in IUCr journals

A basic structural check has been run on your CIF. These basic checks will be run on all CIFs submitted for publication in IUCr journals (*Acta Crystallographica*, *Journal of Applied Crystallography*, *Journal of Synchrotron Radiation*); however, if you intend to submit to *Acta Crystallographica Section C* or *E* or *IUCrData*, you should make sure that full publication checks are run on the final version of your CIF prior to submission.

Publication of your CIF in other journals

Please refer to the *Notes for Authors* of the relevant journal for any special instructions relating to CIF submission.

PLATON version of 19/02/2022; check.def file version of 19/02/2022

Datablock ab1401 - ellipsoid plot

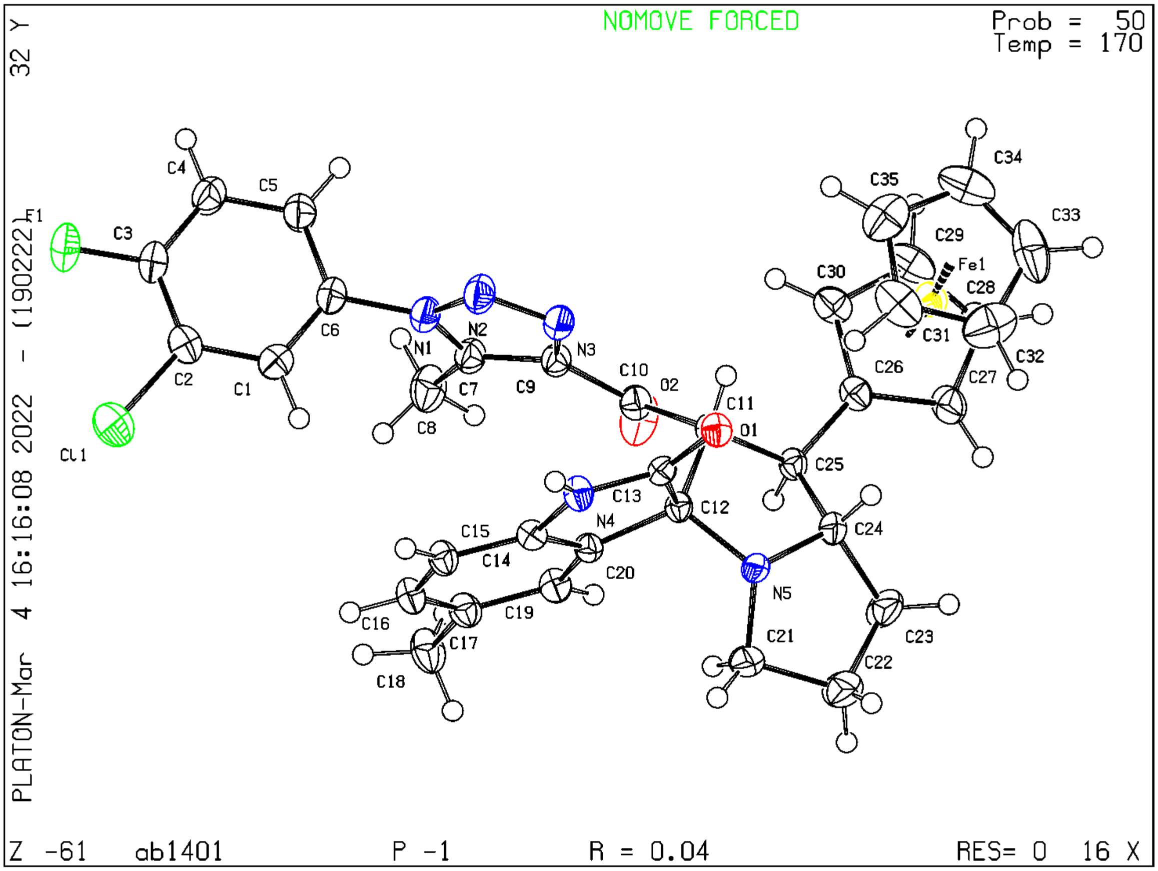

Supplement: Supplementary file 1 [file molecules-27-04095-s001.zip › molecules-1789237-supplementary.pdf]
